# Imitation-relaxation reinforcement learning for sparse badminton strikes via dynamic trajectory generation

**DOI:** 10.3389/fnbot.2025.1649870

**Published:** 2025-09-02

**Authors:** Yanyan Yuan, Yucheng Tao, Shaowen Cheng, Yanhong Liang, Yongbin Jin, Hongtao Wang

**Affiliations:** ^1^Center for X-Mechanics, Zhejiang University, Hangzhou, China; ^2^ZJU-Hangzhou Global Scientific and Technological Innovation Center, Zhejiang University, Hangzhou, China; ^3^State Key Laboratory of Fluid Power and Mechatronic System, Zhejiang University, Hangzhou, China; ^4^Institute of Applied Mechanics, Zhejiang University, Hangzhou, China

**Keywords:** reinforcement learning, robotic badminton, sparse reward, nonlinear dynamics, state prediction, trajectory generation

## Abstract

Robotic racket sports provide exceptional benchmarks for evaluating dynamic motion control capabilities in robots. Due to the highly non-linear dynamics of the shuttlecock, the stringent demands on robots' dynamic responses, and the convergence difficulties caused by sparse rewards in reinforcement learning, badminton strikes remain a formidable challenge for robot systems. To address these issues, this study proposes DTG-IRRL, a novel learning framework for badminton strikes that integrates imitation-relaxation reinforcement learning with dynamic trajectory generation. The framework demonstrates significantly improved training efficiency and performance, achieving faster convergence and twice the landing accuracy. Analysis of the reward function within a specific parameter space hyperplane intuitively reveals the convergence difficulties arising from the inherent sparsity of rewards in racket sports and demonstrates the framework's effectiveness in mitigating local and slow convergence. Implemented on hardware with zero-shot transfer, the framework achieves a 90% hitting rate and a 70% landing accuracy, enabling sustained humanrobot rallies. Cross-platform validation using the UR5 robot demonstrates the framework's generalizability while highlighting the requirement for high dynamic performance of robotic arms in racket sports.

## 1 Introduction

Interceptive robotic ball sports, including table tennis (Büchler et al., 2022), badminton (Mori et al., 2019), and tennis (Zaidi et al., 2023; Hattori et al., 2020) have served as critical testbeds for evaluating the dynamic performance of robotstasks that remain challenging even for skilled human players. These sports typically involve three stages: (1) ball trajectory prediction, (2) hitting strategy decision, and (3) robotic arm motion control. Accurate ball trajectory prediction is crucial for successful interception, while the hitting decision determines the post-impact trajectory and landing position. Real-time motion control ensures the racket reaches the desired state for an effective strike. Collectively, these stages impose stringent demands on precise trajectory prediction, robust hitting decisions, real-time motion control, and high dynamic responsiveness, underscoring the inherent challenges of fast-paced racket sports.

Accurate trajectory prediction intuitively relies on precise dynamics models for ball sports [table tennis (Zhao et al., 2015; Mülling et al., 2010), badminton (Waghmare et al., 2016)]. However, uncertainties in physical parameters often lead to substantial prediction errors, particularly pronounced in badminton due to its significant aerodynamic drag effects and extended flight trajectories (Cohen et al., 2014; Cohen and Clanet, 2016). While Kalman Filter-based approaches have been adopted to enhance accuracy in table tennis (Tebbe et al., 2019, 2021), tennis (Zaidi et al., 2023), badminton (Zhi et al., 2022; Yang, 2022), and other ball (Yu et al., 2023; Hsiao and Kao, 2023) sports. Many approaches simplify aerodynamics by neglecting nonlinear forces (air drag force or Magnus force) or assuming constant aerodynamic coefficients. These simplifications are particularly inadequate for precise badminton trajectory prediction, whose unique feathered structure results in highly nonlinear dynamics and varying drag coefficient due to feather deformation, leading to pronounced velocity decay (initial-to-terminal ratio up to 17.5; Cohen et al., 2014), 5–10 times greater than others. These distinctive characteristics severely complicate accurate state prediction, posing unique challenges, especially over longer horizons.

Successful ball-hitting in robotic racket sports necessitates accurate hitting decisions and real-time motion control. Early approaches utilized collision and trajectory prediction models for calculating the desired racket state (Yu et al., 2023; Tebbe et al., 2019) and trajectory optimization (Yu et al., 2023; Müller et al., 2011) for motion control, which is inherently limited by model parameter accuracy, particularly critical in the badminton task due to their highly nonlinear dynamics and variable drag coefficients. Recent advancements have employed reinforcement learning (DDPG Tebbe et al., 2021; Gao et al., 2022, TD3 Gao and Zell, 2023), evolutionary search (D'Ambrosio et al., 2023; Gao et al., 2020) for hitting decision or jointly learning hitting strategies and joint-level motion control (Abeyruwan et al., 2023; D'Ambrosio et al., 2024), and achieving diverse table tennis playing styles. However, RL approaches often rely on sparse reward functions where the agent rarely sees a reward signal with random exploration (Nair et al., 2018). This inherent sparsity often impedes efficient exploration and leads to convergence to suboptimal policies, thereby requiring considerable iterative sim-to-real training. Learning from Demonstration (LfD) (Muelling et al., 2010; Chen et al., 2021; Akrour et al., 2018; Huang et al., 2016) mitigates this by imitating human behaviors from expert demonstrations, but it typically requires expensive datasets, and its generalization is inherently limited by the provided demonstrations.

Beyond control strategies, the dynamic capabilities of the robotic arm are also crucial. Commercial collaborative robotic arms are commonly employed, particularly in table tennis (Tebbe et al., 2021; Gao et al., 2022; Abeyruwan et al., 2023; Ding et al., 2022), where the required racket speeds and motion ranges are relatively low. However, as the fastest racket-based projectile sport with recorded smash velocities exceeding 137 m/s (Records, 2014), badminton imposes substantially more stringent requirements on the dynamic performance of robotic armsparticularly in joint velocity (>24 rad/s) and acceleration (>600 rad/s^2^) (Rambely and Osman, 2005) for competitive-level returnscompared to other robotic ball sports. To address these challenges, Mori et al. (2019, 2018) developed a lightweight, high-speed robotic arm with pneumatic actuators, achieving a hitting success rate of 69.7%. Meanwhile. Yuan et al. (2025) also highlighted the critical impact of dynamic performance for badminton robots.

To address the convergence challenges of RL due to sparse rewards and the trajectory prediction challenges posed by the highly nonlinear dynamics in badminton, this letter proposes a learning framework (DTG-IRRL) and a robot-badminton system for sparse robotic badminton striking, which integrates the imitation relaxation reinforcement learning (IRRL; Jin et al., 2022) with the dynamic trajectory generation (DTG). The DTG generates a feasible arm reference trajectory as an initial hitting strategy through the prediction results of the shuttlecock's hitting time and point using the initial 10 frames of ball state, analogous to feedforward control. Then, the IRRL stage trains the arm motion controller, leveraging the generated reference trajectory as imitation targets. Exploiting the unimodal characteristics of the imitation reward function and dynamic reference trajectory adjustment of DTG, this framework significantly mitigates the challenges of convergence to local optima and slow convergence due to sparse reward and improves landing accuracy compared to baseline methods.

**Figure 1:**
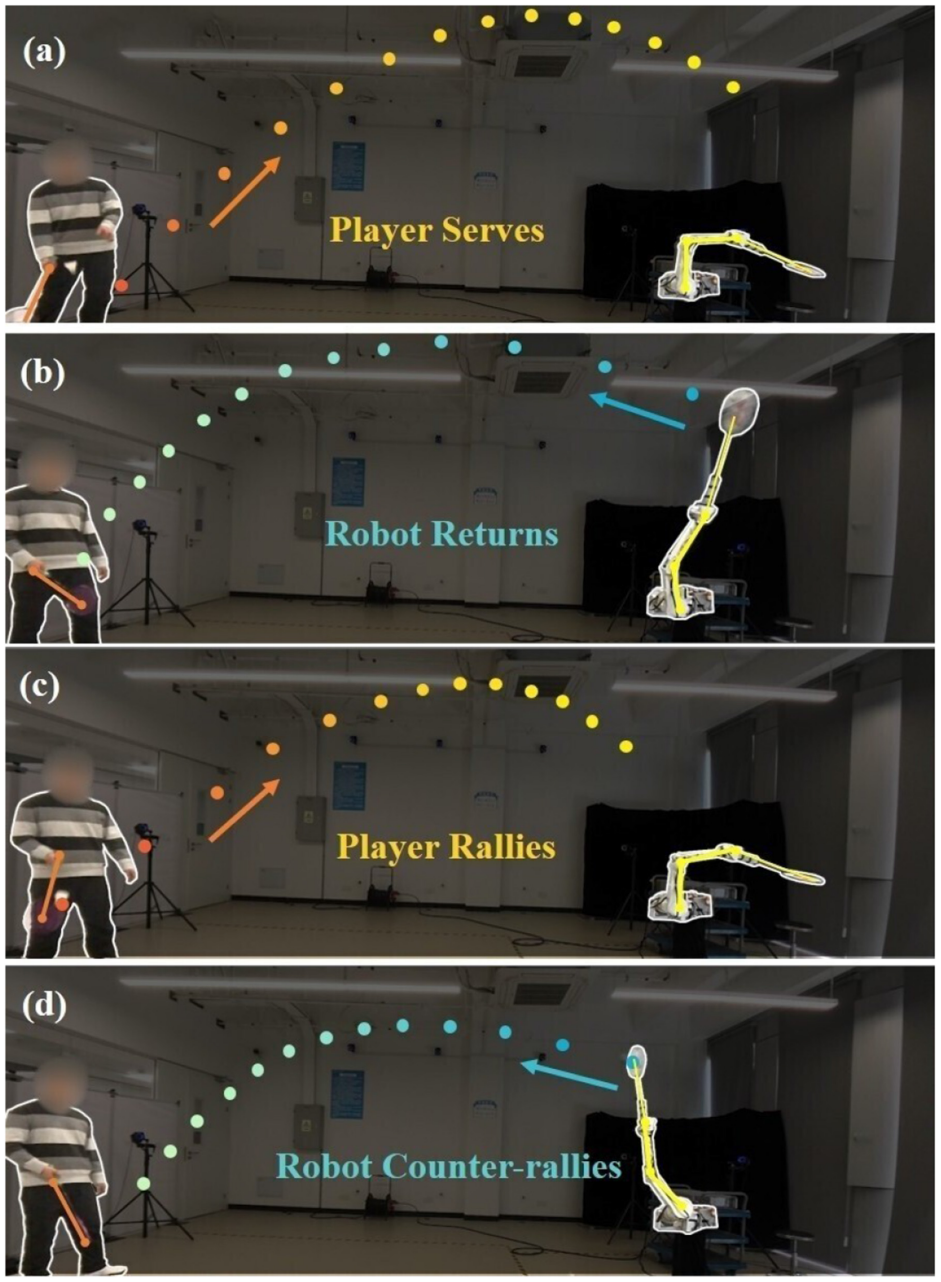
Multi-round human-robot rallies in badminton tasks. **(A)** depicts the player serving; **(B)** shows the robot returning; **(C)** illustrates the player rallying; **(D)** features the robot counter-rallying. The orange and blue dots show the trajectory of the ball during the rally. The yellow and orange lines are the configuration of the robotic arm and human's racket.

The framework has been experimentally validated through hardware implementation with zero-shot transfer. This system includes a 180 Hz motion capture system with ±0.02mm spatial resolution (FZMotion, 2025), a 4-DOF robotic arm exhibiting 234 m/s^2^ peak end-effector acceleration, and a ball launcher. The project is available on https://stylite-y.github.io/DTG-IRRL-For-Badminton/. The key contributions include:

- We propose a learning framework (DTG-IRRL) that integrates imitation relaxation and reinforcement learning with dynamic trajectory generation, achieving faster convergence speeds and a higher success rate. An analysis of the reward distribution in a specific hyperplane intuitively demonstrates the framework's effectiveness in mitigating local and slow convergence due to sparse rewards.
- Hardware implementation on physical platforms with zero-shot transfer demonstrates a 90% hitting rate and 70% landing accuracy, while enabling sustained human-robot rallies exceeding six consecutive strokes, as shown in Figure 1.
- Through comparative evaluations on the UR5 and KirinArm robotic arms, we validate the framework's cross-platform generalization and reveal the impact of robot arm dynamic performance on high-speed ball motion.

## 2 Methodology

### 2.1 Overview

**Figure 2:**
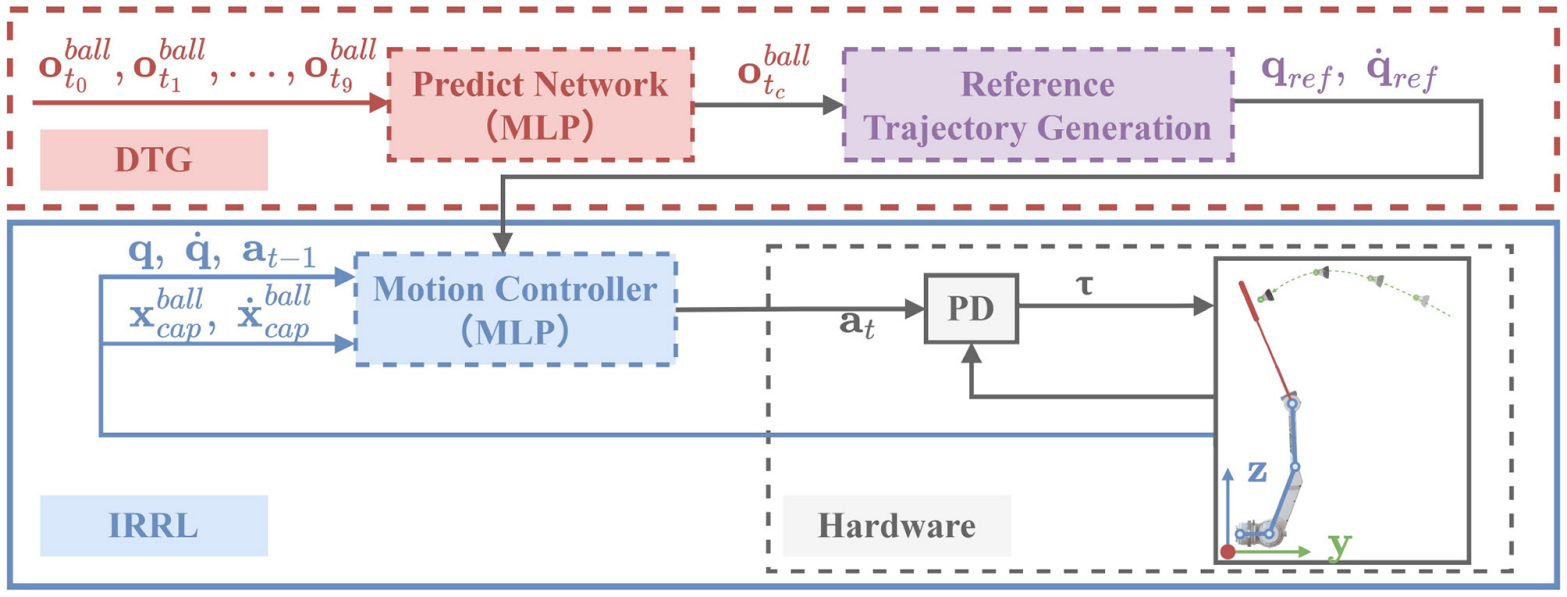
Overview of the DTG-IRRL framework. Prediction Network (red box): Predicts hitting time and the point of the shuttlecock at the target plane based on initial 10-frame observations; Reference Trajectory Generation (purple box): generates the arm's reference trajectory using the prediction results; IRRL (blue box): trains the motion controller by tracking the generated reference trajectory based on the states of both the shuttlecock and the robotic arm.

This section details the proposed learning framework (DTG-IRRL) for real-time badminton striking, illustrated in Figure 2. The framework consists of two components: dynamic trajectory generation (DTG) for robotic arm's reference trajectory generation and imitation relaxation reinforcement learning for motor controller training. Inspired by prior work (Jin et al., 2022), exploiting the unimodal characteristics of the imitation reward function, the IRRL method can guide the policy toward rapid and efficient convergence to mitigate the convergence challenges caused by sparse rewards. However, it typically relies on fixed references, limiting its adaptability for highly dynamic tasks. DTG-IRRL addresses this by dynamically generating a trajectory using a supervised prediction network and a reference trajectory generation module. The motion controller is trained by leveraging the generated trajectory as mimic targets in the imitation phase. The controller is then fine-tuned via reinforcement learning using task-specific rewards in the relaxation phase.

### 2.2 Dynamic trajectory generation (DTG)

#### 2.2.1 Hitting time and point prediction network

The distinctive feathered design of shuttlecocks results in a non-parabolic flight trajectory governed by Tartaglia's curve. The shuttlecock's motion is governed by a nonlinear dynamic system that incorporates gravitational and velocity-dependent aerodynamic drag forces (Cohen et al., 2014; Cohen and Clanet, 2016), as detailed in Equation 1. In simulation, the velocity-related air drag term (second term on the right side of the Equation 1) is also applied to the shuttlecock to simulate the real-world dynamics at each step except for gravity. And *M*, ρ, *C*_*D*_, *R, U* respectively denote shuttlecock mass, air density, drag coefficient, geometric radius, and speed magnitude, while **g**, **U** represent the gravitational acceleration vector and the instantaneous velocity vector.


(1) $$\begin{array}{l} M\frac{d\text{U}}{dt}=M\text{g}-\frac{1}{2}\rho C_{D}\pi R^{2}U\text{U} \end{array}$$

Using initial 10-frame sequences of shuttlecock observations $\left\{{\text{o}}_{t_{0}}^{ball},{\text{o}}_{t_{1}}^{ball},\ldots ,{\text{o}}_{t_{9}}^{ball}\right\}$, we predict the hitting point and time ${\text{o}}_{t_{c}}^{ball}$ of the shuttlecock at user-defined hitting planes (y=0.25) based on the supervised learning network (Figure 2), where ${\text{o}}_{t_{i}}^{ball}=\left\{{\text{x}}_{t_{i}},{\overset{∙}{\text{x}}}_{t_{i}},t_{i}\right\},{\text{o}}_{t_{c}}^{ball}=\left\{{\text{x}}_{t_{c}},t_{c}\right\}$, and ${\text{x}}_{t_{i}},{\overset{∙}{\text{x}}}_{t_{i}},t_{i}$ denote the 3D position and linear velocity of the shuttlecock and the elapsed time since service at the i-th frame, respectively; The network outputs **x**_*t*_*c*__, *t*_*c*_ denote hitting point and time of the ball at the user-defined plane.

The training dataset was generated in a simulation. In badminton flight trajectory, shuttlecocks unique feather structure causes its posture to flip, oscillate, and subsequently stabilize within approximately 130 ms after impact (Cohen et al., 2014), leading to variations in the drag coefficient. Therefore, we implement domain randomization to randomly sample the drag coefficient between [0.62, 0.69] during the initial 130 ms of flight for each simulation cycle and hold it constant thereafter in both the prediction network training datasets collection and the RL training process to capture the inherent nonlinear dynamics of the shuttlecock. The prediction network employs an MLP network with two hidden layers of 256 units in each. Training minimizes the mean squared error (MSE) between the predicted and actual hitting point and time.

#### 2.2.2 Reference trajectory generation

Simultaneously learning both hitting strategy and motion control using RL is challenging due to the sparse reward function. To mitigate this, we calculate the robotic arm's reference trajectory based on trajectory prediction results via reference trajectory generation, which serves as an initial hitting strategy. This strategy implicitly provides initial solutions for desired racket orientation, position, and hitting time, analogous to feedforward control. This initial guidance significantly improves training efficiency and accelerates convergence.

We employed a sigmoid-based trajectory (Equation 2) that guarantees C^2^ continuity in joint space, preventing torque fluctuations. For the 4-DOF robotic arm, target joint angles Θ^*tar*^ ∈ ℝ^4^ can be computed via inverse kinematics from ${\text{o}}_{t_{c}}^{ball}$, with the wrist pitch angle fixed for strike consistency. The reference trajectory is fully determined by three parameters: initial joint angles Θ^*init*^, target angles Θ^*tar*^, and hitting time *t*_*c*_, shown in Equation 3, where *a*_*i*_, *b*_*i*_, *c*_*i*_, *d*_*i*_ are the parameters of the function.


(2) $$\begin{array}{l} \theta_{i}\left(t\right)=\{\begin{array}{c} \theta_{i}^{init},\text{ if t}\leq 0.02\text{ s}\\ \frac{a_{i}}{1+e^{-b_{i}\left(t-c_{i}\right)}}+d_{i}, \end{array}\;i=\left\{1,2,3,4\right\} \end{array}$$

(3) $$\begin{array}{l} \{\begin{array}{l} a_{i}=2\left(\theta_{i}^{tar}-\theta_{i}^{init}\right)\\ b_{i}=20\left(1-t_{c}\right)\\ c_{i}=t_{c}-0.1\\ d_{i}=\theta_{i}^{init} \end{array} \end{array}$$

### 2.3 Imitation-relaxation reinforcement learning (IRRL)

Leveraging the generated trajectory as imitation targets, the two-stage imitation-relaxation reinforcement learning framework (Jin et al., 2022) is employed to accelerate policy convergence and avoid local optima. In the imitation stage, the policy learns to mimic trajectories generated by the DTG module (shown in Figure 2). In the relaxation stage, policies are refined through reward shaping to achieve precise striking and landing control.

#### 2.3.1 Training environment

To learn the badminton striking strategy, we model the task as a finite-horizon, discounted Markov Decision Process (MDP) M(S,A,P,R,γ), where S,A,γ is the state space, action space, and discount factor, and P denotes the state transition dynamics: S×A→S, and each transition is rewarded with a reward function *r*: S×A→R. We use MuJoCo as the physics engine for simulation. The policy, represented by an MLP network with two hidden layers of 512 units in each, was trained with the PPO algorithm (Schulman et al., 2017) in the Stable Baselines3 package.

#### 2.3.2 State and action

The state *s* is an 18-dimensional vector defined as $\text{s}={\left[\text{q},\overset{∙}{\text{q}},\text{x},\overset{∙}{\text{x}},{\text{a}}_{t-1}\right]}^{T}$, where $\text{q},\overset{∙}{\text{q}}\in {\mathbb{R}}^{4},\text{x},\overset{∙}{\text{x}}\in {\mathbb{R}}^{3},{\text{a}}_{t-1}\in {\mathbb{R}}^{4}$ are the joint angle and velocity of the robotic arm, ball position and linear velocity, and previous action. The real-time shuttlecocks position and velocity are received as feedback state to compensate for the prediction deviations caused by the nonlinear dynamics and variations in drag coefficient. In simulation, the initial position and velocity range of the badminton are as follows: *x* ∈ [−0.12, −0.14], *y* ∈ [3.8, 4.2], *z* ∈ [0.6, 0.8], *V*_*x*_ ∈ [−0.8, 0.8], *V*_*y*_ ∈ [−10, −7.5], *V*_*z*_ ∈ [6.0, 7.5]. The action space **a** is the desired joint angle. To facilitate learning, we train the policy to infer the desired angle around the default angle of the robotic arm θ_*default*_. Therefore, the desired joint angle can be calculated by


(4) $$\begin{array}{l} \theta_{des}=\theta_{default}+k{\text{a}}_{t} \end{array}$$

#### 2.3.3 Imitation stage reward

For the imitation stage, the policy learns to track reference trajectories through a composite reward function consisting of the joint and velocity trajectory imitation term *r*^*m*^, the action smoothness and power penalty term *r*^τ^, and the collision penalty *r*^*c*^. Each reward is shaped using a Gaussian kernel and normalized to (0, 1), as shown in Equation 5. **ω** = [ω_*m*_, ω_τ_, ω_*c*_, ω_*sp*_] is the reward weight coefficient. For all undesirable interactions excluding robot-ball contact and ball-ground impact, *r*^*c*^ will return a negative value of −1.


(5) $$\begin{array}{l} \;r_{t}=\omega_{m}r^{m}+\omega_{\tau }r^{\tau }+\omega_{c}r^{c}\\ r^{m}=1.75e^{-\|(q-q_{ref})/q_{m}{\|}^{2}}+0.75e^{-\|(\dot{q}-{\dot{q}}_{ref})/{\dot{q}}_{m}{\|}^{2}}\\ r^{\tau }=2.0e^{-\|(\tau \dot{q})/(\tau_{m}{\dot{q}}_{m}){\|}^{2}}+\\ \;1.0e^{-\|a_{t}-a_{t-1}{\|}^{2}}+1.0e^{-0.5\|a_{t}-2a_{t-1}+a_{t-2}{\|}^{2}}\\ r^{c}=\begin{array}{cc} 0, & \text{if no collision}\\ -1, & \; \end{array} \end{array}$$

#### 2.3.4 Relaxation stage reward

For the relaxation stage, the sparse task-specific rewards *r*^*sp*^ are introduced to guide the policy in achieving badminton striking. *r*^*sp*^ contains two components: *r*^*h*^ and *r*^*l*^, where *r*^*h*^ is activated upon racket-shuttlecock contact, providing a positive reward, and *r*^*l*^ is triggered when the shuttlecock lands within the target area, yielding a positive reward. The total reward shown in Equation 6 is employed to train the policy in the relaxation stage.


(6) $$\begin{array}{l} \;r_{t}=\omega_{m}r^{m}+\omega_{sp}r^{sp}+\omega_{\tau }r^{\tau }+\omega_{c}r^{c}\\ r^{sp}=250r^{h}+250r^{l}\\ \;r^{h}=\begin{array}{cc} 1, & \text{if racket collides with ball}\\ 0, & \; \end{array}\\ \;r^{l}=\begin{array}{cc} 1, & \text{if ball lands in target area}\\ 0, & \; \end{array} \end{array}$$

### 2.4 Domain randomization

To enhance the robustness of the policy trained in simulation and overcome the sim-to-real gap, we implement domain randomization (Hwangbo et al., 2019) for the kinematic and dynamic parameters of both the robotic arm and the shuttlecock. We randomize the initial angle, PD gains of the robotic arm, initial position and velocity of the ball, air drag coefficient, and system delay at the beginning of each rollout. Meanwhile, because the motion capture system introduces an approximate delay of 10 ms, we introduce a random delay of 0–20 ms to the shuttlecock's state observation during the training stage to enhance the controller's robustness to sensor latency. Additionally, noise perturbations are introduced to both network observations and robotic arm joint torques to enhance perceptual realism and controller robustness. The ranges for randomization of each parameter and noise are specified in Table 1.

**Table 1:** Domain randomization ranges.

**Parameters**	**Range**	**Unit**
Kp factor	[0.9, 1.1]	/
Kd factor	[0.9, 1.1]	/
Initial Joint angle rand	[−0.1, 0.1]	*rad*
Initial ball position rand	[−0.1, 0.1]	*m*
Air drag coefficient	[0.65, 0.69]	/
System delay	[0, 20]	*ms*
Initial ball velocity	*v*_*x*_ = [−0.8, 0.8]	*m*/*s*
	*v*_*y*_ = [−10.0, −7.5]	
	*v*_*Z*_ = [6.0, 7.0]	

## 3 Experiment and results

### 3.1 Experiment platform

#### 3.1.1 Robot-badminton system

**Figure 3:**
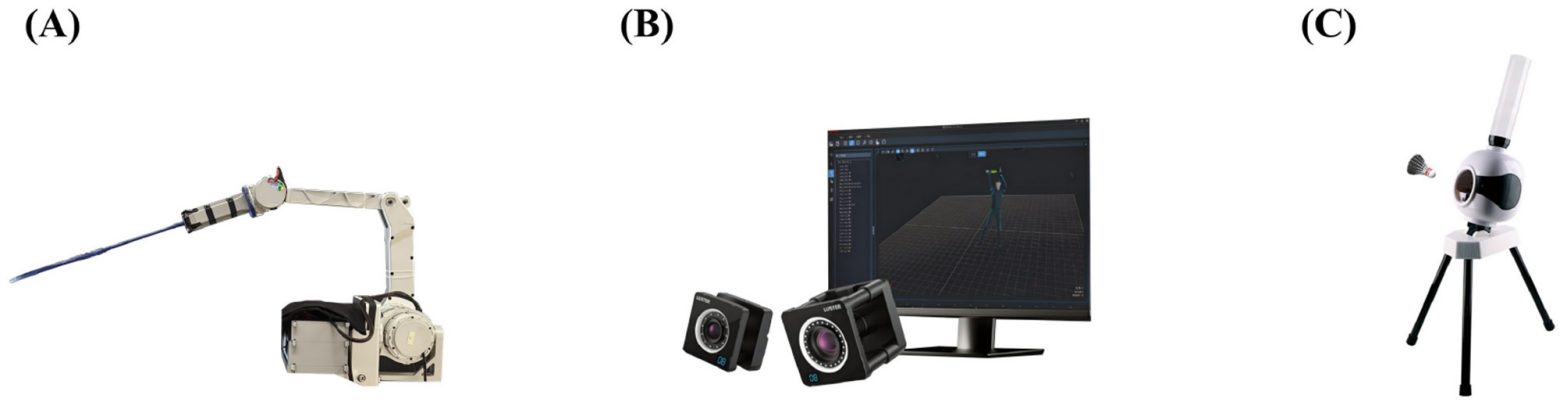
**(A)** The 4-DOF robotic arm named KirinArm. **(B)** Motion capture system. **(C)** Shuttlecock launcher.

The proposed framework is implemented on a high-dynamic 4-DOF robotic arm (KirinArm) with a 500 Hz control frequency, as shown in Figure 3A. A programmable shuttlecock launcher is implemented, featuring speed control in the range of 5–30 m/s and an adjustable angle of ±30° (Figure 3C).

#### 3.1.2 Motion capture system

The FZMotion optical motion capture system (FZMotion, 2025) is deployed for real-time shuttlecock tracking, using a 16-camera array operating at 180Hz sampling frequency with 2048 × 1536 resolution (Figure 3B).

#### 3.1.3 Simulation platform

The policy is trained on a workstation with an AMD Ryzen Threadripper 3970X @ 2.20 GHz, and an NVIDIA RTX 3090Ti GPU. The policy achieved convergence within approximately 10 hours through parallel training across 60 environments for 36,000 iterations. The physical simulation frequency and control frequency are 1000 Hz and 500 Hz, respectively. The policy is training with weight **ω** = [0.4, 0.4, 0.1, 0.1]. The detailed hyperparameters are shown in Table 2.

**Table 2:** Hyperparameters for PPO and neural network.

**Parameters**	**Value**
Number of environments	60
Learning epochs	8
Learning rate	0.0001
Gamma	0.995
Lamda	0.99
Number of batches	4
Network hidden layers	[512, 512]

### 3.2 Shuttlecock prediction results analysis

The accuracy of the prediction network determines the initial reference trajectory precision, while its inference time affects training efficiency. To train the prediction network, we generated 22,000 shuttlecock trajectories with randomized initial positions and velocities in simulation to ensure data diversity. For real-world validation, 206 sets of real trajectories were collected, with the first 10 frames of each serving as input to test the shuttlecocks hitting point and time error at the user-defined plane.

The proposed prediction network achieved average position errors of 0.040 m (X) and 0.078 m (Z), with a 0.024 s time error. In comparison, the physics-based model (Waghmare et al., 2016) exhibited prediction errors approximately 4-fold larger (0.21m in X, 0.26m in Z), while an EKF-RBF method (Zhi et al., 2022) yielded landing position prediction errors of 0.08m (X) and 0.15 m (Y), exhibiting maximum errors 2 times that of ours. The network trained on simulated data with a fixed drag coefficient (Yang, 2022) resulted in an average spatial error of 0.13 m, 1.5 times that of ours. These results demonstrate the superior accuracy of the prediction network on the highly nonlinear dynamics. Furthermore, we conducted 100 trials to assess the computational cost of the reference trajectory generation module, which impacts the policy training efficiency. The average cost is 0.048 ms, significantly below the 2 ms control cycle.

It is worth noting that in the DTG-IRRL framework, the prediction network (red dotted frame in Figure 2) serves to provide an a priori estimate of the shuttlecock's interception point. This estimate is used exclusively during the training phase to generate an initial reference trajectory for the robot arm. Crucially, the DTG module itself is a training-time component designed to guide the controller to learn the motion style of the reference trajectory and is not deployed on the hardware. The final policy deployed on the physical robot is solely the motion controller (as depicted in the blue frame in Figure 2). Furthermore, during simulation, the prediction module operates independently for each shot, eliminating drift issues.

### 3.3 DTG-IRRL framework training results analysis

To validate the effectiveness of the DTG-IRRL framework for badminton striking, we conducted a comparative analysis across three training paradigms:

- **DTG-IRRL**: integrates IRRL with the DTG module.
- **IRRL-o**: utilizes IRRL without the DTG module, relying on a fixed reference trajectory independent of the shuttlecock's state.
- **DTG-RL**: one-stage RL with DTG module, where the policy is trained directly via reward Equation 6 without an imitation learning stage.

All implementations maintained the identical network architectures and hyperparameters.

**Figure 4:**
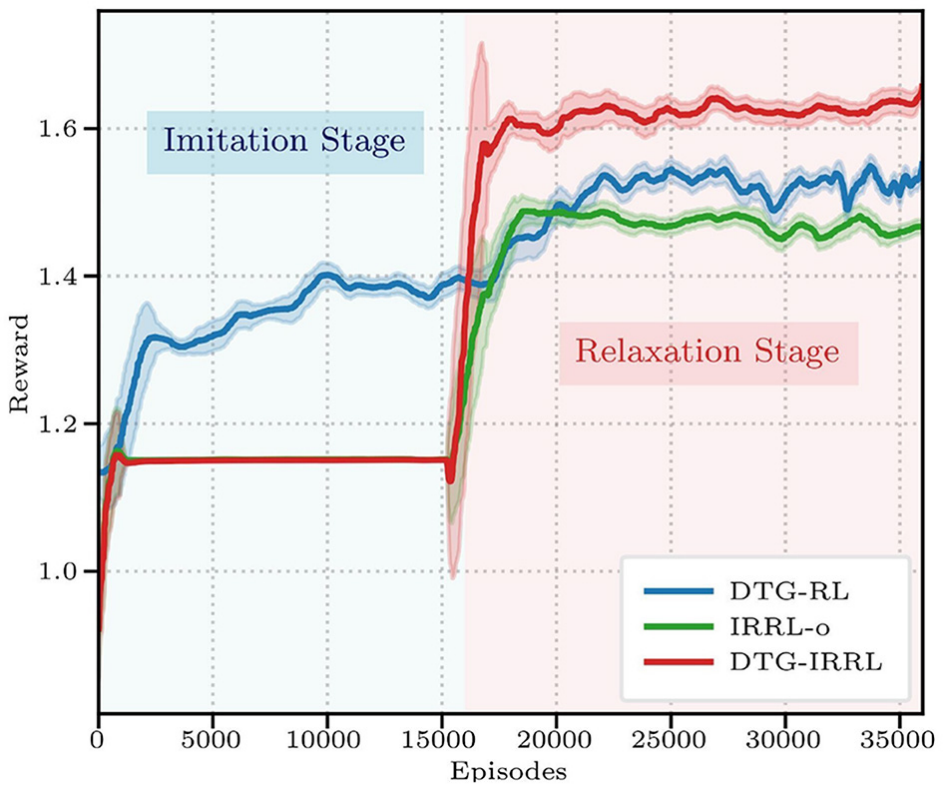
The reward curves of three frameworks: DTG-IRRL (red), DTG-RL (blue), and IRRL (green). The light blue shaded area indicates the imitation training stage, while the light red shaded area denotes the relaxation stage.

Quantitative comparisons (Figure 4) demonstrate DTG-IRRL's superior performance, converging in 20,000 episodes with a peak reward of 1.62 during relaxationoutperforming both DTG-RL (converging in 25,000 episodes with a reward of 1.56) and IRRL-o (1.48). While IRRL-o matches DTG-IRRL's convergence rate, its significantly lower final reward highlights the prediction network's contribution.

**Figure 5:**
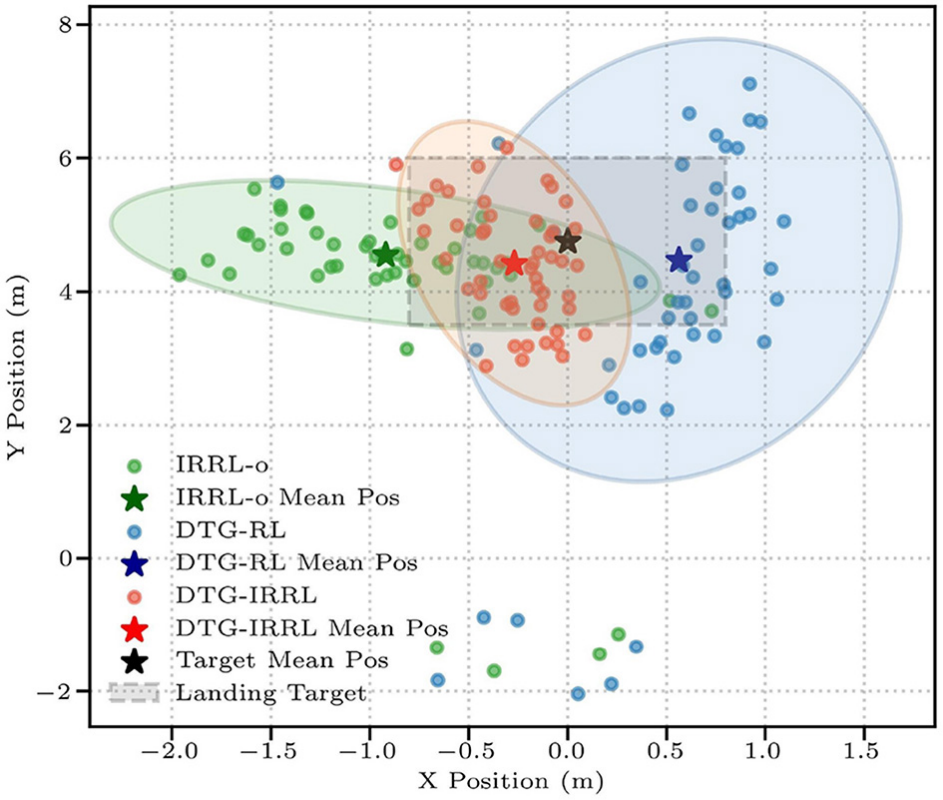
The shuttlecock landing positions across three frameworks: DTG-IRRL (red circle), DTG-RL (blue circle), and IRRL (green circle). The light red, blue, and green shaded areas represent the 90% confidence intervals for landing positions, while the pentagram is the average position of the landing point, and the light gray box denotes the target landing area.

Then we evaluate the policy performance using three metrics: hitting rate (*R*_*h*_), landing accuracy (*R*_*l*_), and landing deviation (*D*_*e*_) (Equation 7) between the average landing position and the target center, where lower values indicate better precision. The target landing area spans *x* ∈ [−0.8, 0.8]*m* and *y* ∈ [3.5, 6.0]*m*, centered at [0.0, 4.75] m. Quantitative evaluation through 50 simulated trials shows DTG-IRRL's superior control performance, achieving 100% *R*_*h*_ with 80% *R*_*l*_ (shown in Table 3). In comparison, IRRL-o and DTG-RL show significantly lower *R*_*l*_ (34% and 36%, respectively). DTG-IRRL achieved superior landing deviation with *D*_*e*_ = 0.426 approximately 67% of DTG-RL's (*D*_*e*_ = 0.63) and half of IRRL-o's (*D*_*e*_ = 0.0.94), illustrated in Figure 5. Notably, DTG-IRRL's landing points are tightly clustered within the target zone, whereas DTG-RL produces scattered distributions, and IRRL-o misses the target in 66% of cases. IRRL-o performs poorly in accommodating shuttlecock deviation due to the absence of initial reference trajectory guidance. DTG-RL's lack of imitation learning results in slower convergence and potential convergence to suboptimal policies because of the non-convex reward function – such as the robotic arm adopting unnatural motion patterns like moving quickly to a vertical position, remaining stationary, and swinging just before the ball's arrival.


(7) $$\begin{array}{l} \begin{array}{c} D_{e}=∥{\text{x}}_{land}-{\text{x}}_{tar}{∥}_{2} \end{array} \end{array}$$

Comparative analysis reveals two primary advantages of DTG-IRRL: (1) Leveraging the generated trajectory, the IRRL strategy efficiently achieves faster convergence by exploiting the unimodal nature of the imitation reward function in the parameter space; (2) utilizing the initial arm reference trajectory generated by DTG, DTG-IRRL dynamically adjusts the initial reference trajectory based on the prediction results, thereby enhancing the hitting rate and landing accuracy.

**Table 3:** Results of badminton striking tests.

**Target area (m)**	**Robotic arm**	**Approach**	***R*_*h*_**	***R*_*l*_**	***D*_*e*_ (m)**
*x* ∈ [−0.8, 0.8] *y* ∈ [3.5, 6.0]	**KirinArm**	**DTG-IRRL**	**100%**	**80%**	**0.426**
		DTG-RL	88%	36%	0.63
		IRRL-o	92%	34%	0.94
*x* ∈ [−0.8, 0.8] *y* ∈ [3.5, 6.0]	**KirinArm**	**DTG-IRRL**	**100%**	**80%**	**0.426**
	UR5	DTG-IRRL	94%	58%	1.17
*x* ∈ [−0.8, 0.8] *y* ∈ [5.0, 8.0]	**KirinArm**	**DTG-IRRL**	**96%**	**72%**	**0.59**
	UR5	DTG-IRRL	90%	0.0%	2.75

Bold values indicates the best-performing results obtained using the DTG-IRRL learning framework and the KirinArm robotic arm.

### 3.4 Sparse rewards analysis on hyperplane

**Figure 6:**
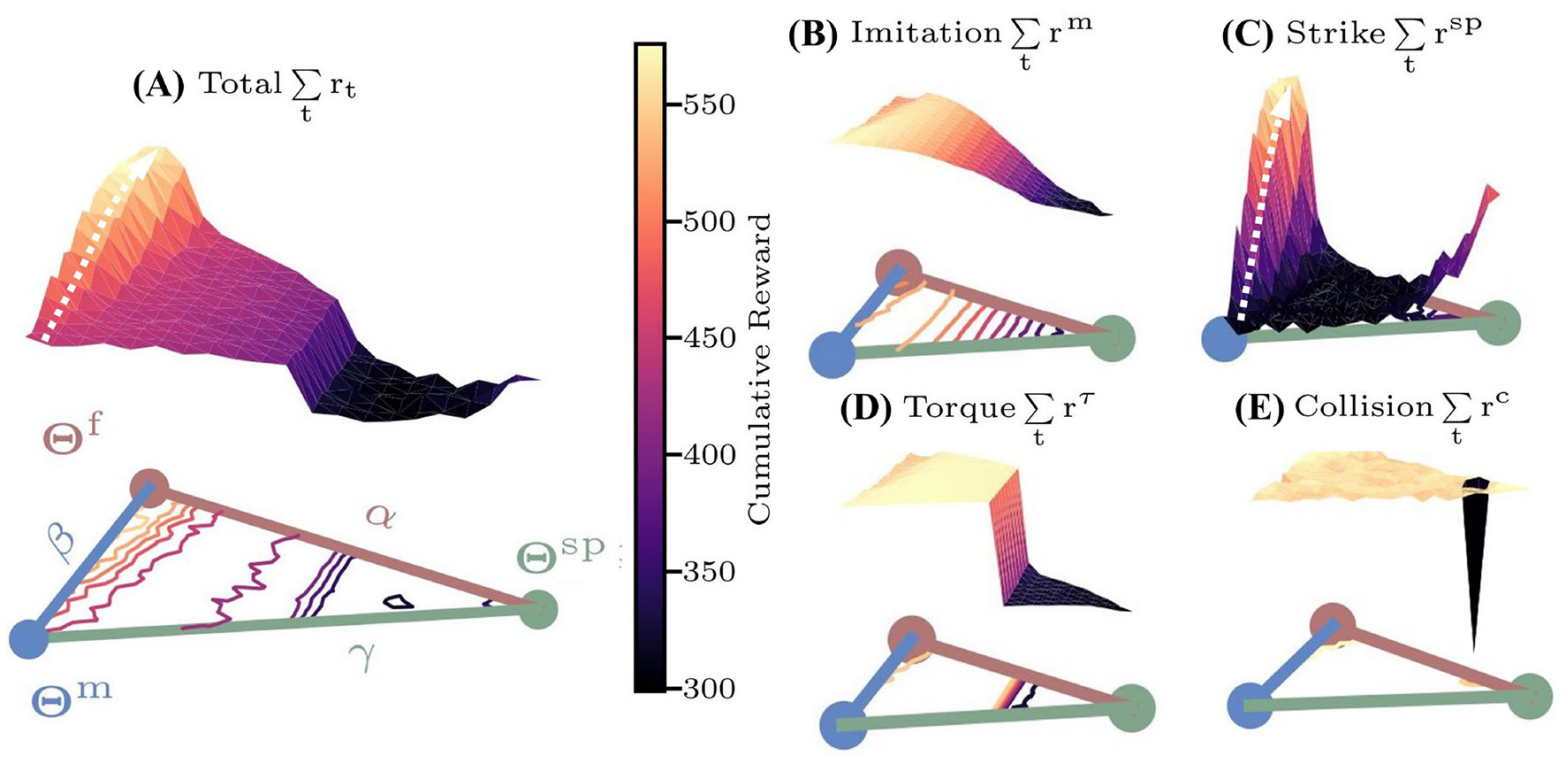
The cumulative reward surfaces over the characteristic hyperplane based on Θ^*f*^, Θ^*m*^, Θ^*sp*^. Colored lines within the plane represent contour lines. **(A)** Total reward *r*_*t*_; **(B)** imitation reward *r*^*m*^; **(C)** sparse reward *r*^*sp*^; **(D)** torque reward *r*^τ^; **(E)** collision reward *r*^*c*^.

For high-dimensional nonlinear multi-objective problems, the policy is sensitive to the distribution of the reward function and the initial solution settings. In racket sports, the sparse reward and the high-dimensional network parameter space complicate policy convergence analysis. Therefore, inspired by Jin et al. (2022), we define a special parameter space hyperplane to analyze the reward function distribution:


(8) $$\begin{array}{l} \Theta^{l}=\alpha \Theta^{f}+\beta \Theta^{m}+\gamma \Theta^{sp}\\ \;\alpha +\beta +\gamma =1\\ \;\alpha ,\beta ,\gamma \geq 0, \end{array}$$

where Θ**^f^**, Θ^**m**^, Θ^**sp**^ are network parameters of the controllers **U**(**x**; Θ^**f**^), **U**(**x**; Θ^**m**^), **U**(**x**; Θ^**sp**^) that are trained using the reward functions with *r*_*t*_, *r*^*m*^ and *r*^*sp*^ in Equations 5, 6, **x** is the input vector. The surface plot of the cumulative reward on the hyperplane can be represented as a ternary plot with *ω* = [0.4, 0.4, 0.1, 0.1] (Figure 6A), where **η** = (α, β, γ) represent the triangle coordinates of Θ^**l**^. The reward for each controller is averaged over 50 simulation trials.

Results in Figure 6C indicate that the sparse reward surface $r_{t}=r^{sp}$ exhibits multi-local optima on the hyperplane, with a global maximum around Θ^**f**^, where the policy initialized around Θ^**sp**^ will converge to the suboptimal solution Θ^**sp**^. Furthermore, the gradients of the reward surface are low in regions distant from the three special controllers (black area in Figure 6C), severely hindering convergence speed. In contrast, the torque and collision reward surface (Figures 6D, E) both have a single maximum. And the imitation reward surface $r_{t}=r^{m}$ (Figure 6B) features a single maximum near Θ^**m**^, enabling fast convergence from any initial **U**(**x**; Θ). Notably, both sparse and total reward (Figure 6A) surfaces exhibit ascending gradients from Θ^**m**^ to Θ^**f**^, suggesting that initializations around Θ^**m**^ can effectively guide convergence to the optima Θ^**f**^. Leveraging these characteristics, the DTG-IRRL firstly guides the policy to quickly converge to **U**(**x**; Θ^**m**^) (white arrow in Figure 6B) using only *r*^**m**^. Then, utilizing Θ^**m**^ as the initial parameters, the policy can converge to the optimal controller **U**(**x**; Θ^**f**^) with the total reward *r*_*t*_ (white arrow in Figure 6A). Empirical results demonstrate that DTG-IRRL can effectively mitigate the challenges of convergence to local optima and slow convergence due to sparse reward.

**Figure 7:**
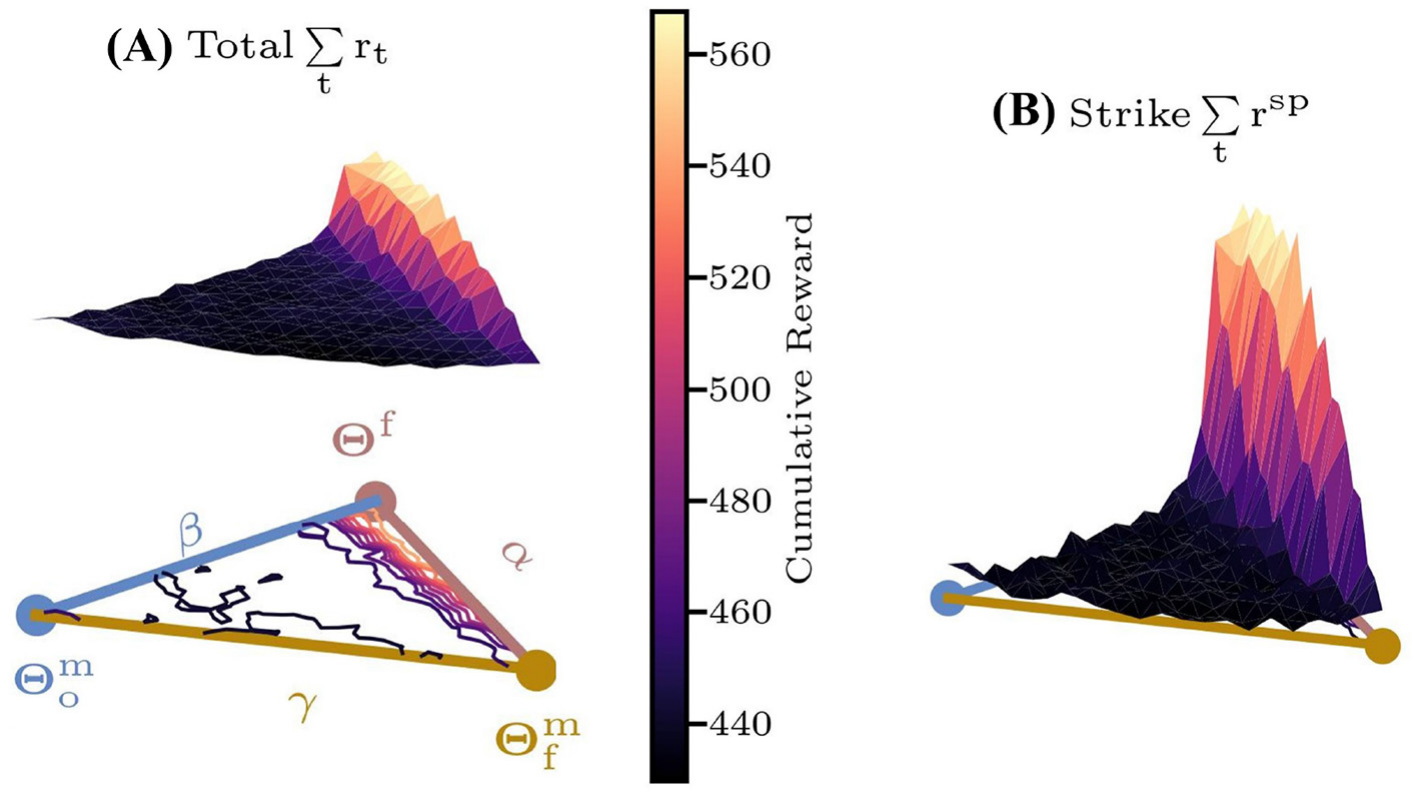
The cumulative reward surfaces over the characteristic hyperplane based on $\Theta^{f},\Theta_{f}^{m},\Theta_{o}^{m}$. Colored lines within the plane represent contour lines. **(A)** Total reward *r*_*t*_; **(B)** sparse reward *r*^**sp**^.

Moreover, to evaluate the impact of the framework's dynamic reference trajectory adjustment on policy performance, we also define a parameter space hyperplane based on $\Theta^{f},\Theta_{f}^{m},\Theta_{o}^{m}$, where $\Theta_{f}^{m},\Theta_{o}^{m}$ denote the parameters of controllers $\text{U}\left(\text{x};\Theta_{f}^{m}\right),\text{U}\left(\text{x};\Theta_{\text{o}}^{\text{m}}\right)$, trained via DTG-IRRL and IRRL-o methods with reward *r*^**m**^, respectively. As illustrated in Figure 7, both the sparse and total reward surfaces reveal that the gradient from $\Theta_{\text{o}}^{\text{m}}$ to optima Θ^**f**^ is notably low in most regions, with significant gradient only near Θ^**f**^. This indicates that IRRL-o converges inefficiently unless initialized close to Θ^**f**^. In contrast, DTG-IRRL demonstrates a pronounced gradient from $\Theta_{\text{f}}^{\text{m}}$ to Θ^**f**^, suggesting that policies initialized around $\Theta_{\text{f}}^{\text{m}}$ can effectively and quickly converge to Θ^**f**^. These results confirm that DTGs dynamic trajectory adjustment capability enables the policy to converge to the optimal solution Θ^**f**^ more efficiently and quickly.

### 3.5 Reward weights sensitivity and policy stability analysis

#### 3.5.1 Reward weights sensitivity analysis

**Figure 8:**
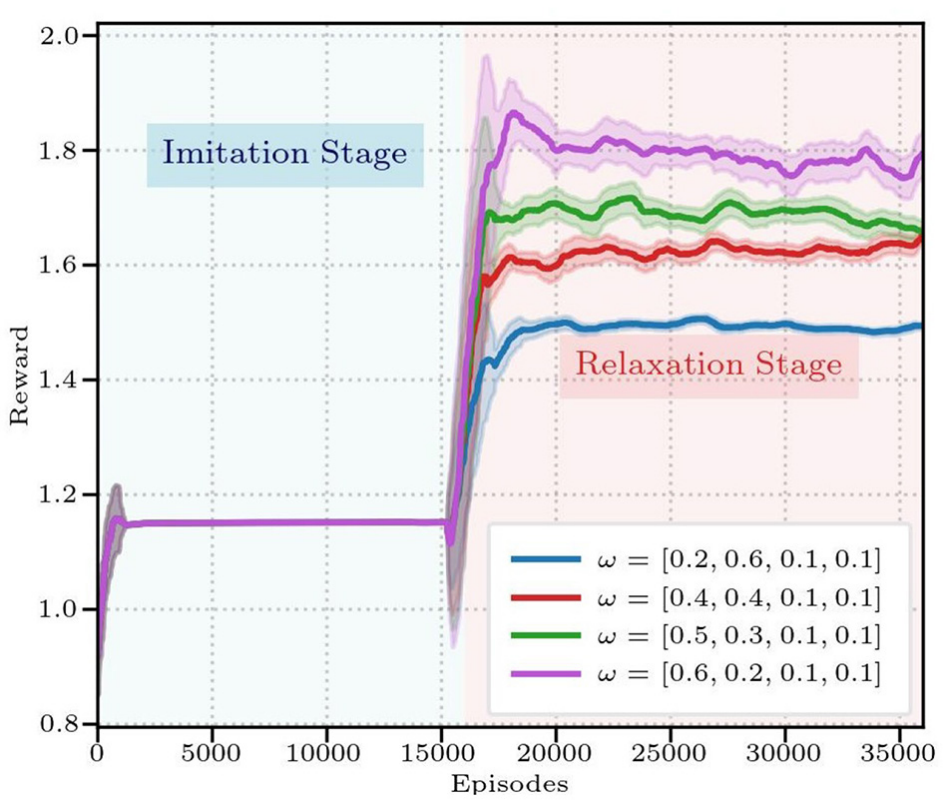
The reward curves of three frameworks under different reward weights.

To further verify the effectiveness of the DTG-IRRL in guiding faster policy convergence and its sensitivity to different weights, we conducted an ablation study to evaluate the impact of the different weights for the imitation and sparse reward components during the relaxation stage of training. We tested four different weight configurations, and the reward curves are presented in Figure 8. The results demonstrate that the policy's convergence is not sensitive to different weights. While the final cumulative reward values differ due to the scaling factor of the sparse reward weights, all four policies exhibit similar convergence profiles, stabilizing after approximately 20,000 training iterations.

To quantitatively evaluate the landing accuracy of different rewards on landing accuracy, each policy was evaluated over 50 hitting trials, with the results summarized in Table 4. The results show that all four policies achieve consistently high performance: the hitting rate (*R*_*h*_) exceeds 90%, landing accuracy (*R*_*l*_) is approximately 80%, and the landing deviation (*D*_*e*_) is around 0.4m. This demonstrates that the final policy's performance is robust to variations in the reward function weights. We attribute this stability to our DTG-IRRL framework, which effectively guides the policy to reach the optimal solution, making it less sensitive to minor tuning of reward components.

**Table 4:** Test results of different reward weights.

**Reward weights**	***R*_*h*_**	***R*_*l*_**	***D*_*e*_(*m*)**
ω = [0.2, 0.6, 0.1, 0.1]	92%	74%	0.37
ω = [0.4, 0.4, 0.1, 0.1]	100%	80%	0.43
ω = [0.5, 0.3, 0.1, 0.1]	98%	74%	0.36
ω = [0.6, 0.2, 0.1, 0.1]	94%	78%	0.40

#### 3.5.2 Stability analysis

**Figure 9:**
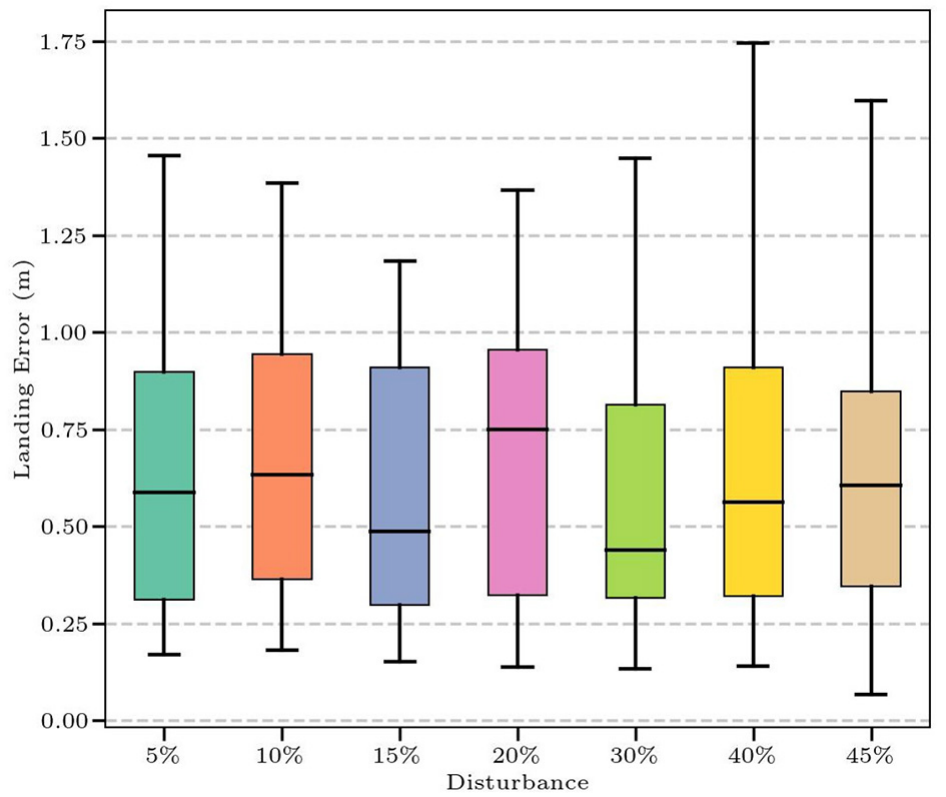
The landing error under different levels of disturbance.

Stability determines the controller's ability to resist external perturbations. However, theoretical stability analysis is often difficult for systems controlled by complex, high-dimensional neural network policies. Therefore, inspired by prior work [1], we conducted an empirical analysis to assess the systems stability to perturbations. We introduced varying levels of disturbance (from 5% to 45%) to the initial position and velocity of the shuttlecock while ensuring the initial states remained within the training distribution (Table 5). We tested the controllers landing error (defined as the Euclidean distance between the landing position and the target center) under different disturbances. The landing box-line error plot in Figure 9 demonstrates that the landing errors of most balls under different disturbances are small. When the disturbance is above 40%, the variance of the landing error increases slightly. The results, shown in Table 5, illustrate remarkable stability. Despite significant perturbations, the hitting rate (*R*_*h*_) remains at 100% and landing accuracy (*R*_*l*_) stays above 94%. Crucially, the landing deviation (*D*_*e*_) shows only a slight fluctuation increase, approximately 2.6%, which can be almost ignored. Within the operational range, the closed-loop system maintains consistent performance despite increasing disturbances, demonstrating inherent stability.

**Table 5:** Test results of different level disturbance.

**Disturbance**	***R*_*h*_**	***R*_*l*_**	***D*_*e*_(*m*)**
5%	100%	94%	0.189
10%	100%	96%	0.183
15%	100%	100%	0.192
20%	100%	96%	0.187
30%	100%	96%	0.184
40%	100%	98%	0.201
45%	100%	94%	0.194

### 3.6 Generalization of DTG-IRRL framework

To validate the generalizability of the framework, we deployed it on a UR5 robotic arm (UR5e, 2025) using identical hyperparameter configurations. Moreover, to analyze the influences on shuttlecock speed and flight distance of the robotic arm's dynamic performance, we trained two controller–one for the UR5 and another for the KirinArm–using the DTG-IRRL framework with identical training parameters, varying only the target landing areas. A multi-rally experiment then compared their performance across different landing distances.

The results shown in Table 3 demonstrate successful generalization across robotic platforms in 50 trials, with both KirinArm and UR5 achieving high *R*_*h*_ (100% and 94%, respectively). *R*_*l*_ was 80% (KirinArm) and 58% (UR5). Quantitative analysis shows that a higher *D*_*e*_ value for UR5 (1.17 m) compared to KirinArm (0.426 m), indicating relatively lower accuracy. Crucially, these results were achieved without a reward function or weight adjustments, demonstrating cross-platform generalization capability.

**Figure 10:**
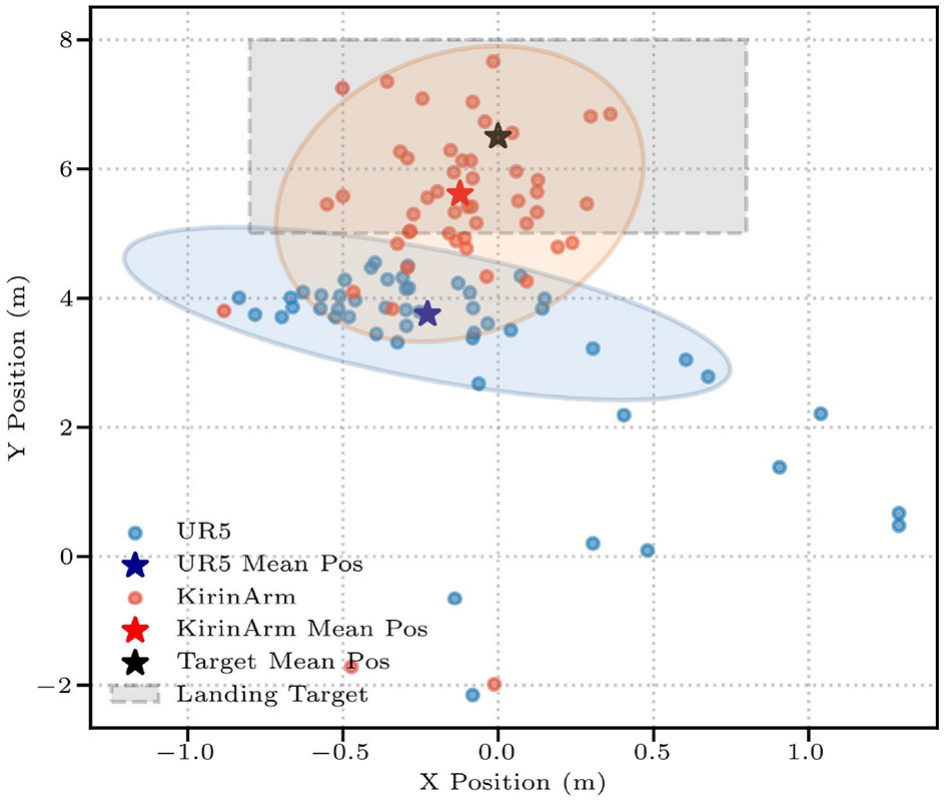
The shuttlecock landing positions of KirinArm (red circle) and UR5 (blue circle). The light red and blue shaded areas represent the 90% confidence intervals for landing positions, while the pentagram is the average position of the landing point; the light gray box denotes the target landing area.

Experimental results in Table 3 reveal significant performance differences between KirinArm and UR5 across varying target distances. For close-range targets, both KirinArm and UR5 achieve *R*_*h*_ exceeding 90% and *R*_*l*_ exceeding 50%. Notably, KirinArm demonstrated superior accuracy, exhibiting a 1.5-fold higher *R*_*l*_ and a 50% reduction in *D*_*e*_ compared to the UR5. At farther ranges, while UR5 maintained an *R*_*h*_ of 90%, it failed to land the shuttlecock within the target area (*R*_*l*_= 0%). In contrast, KirinArm sustains high performance (*R*_*h*_= 96%, *R*_*l*_= 72%). Figure 10 shows landing distributions from 50 trials, highlighting KirinArm's consistent precision with an average position nearer to the target center and a substantially lower *D*_*e*_ (0.59 vs UR5's 2.75, 21.5% of UR5's).

**Figure 11:**
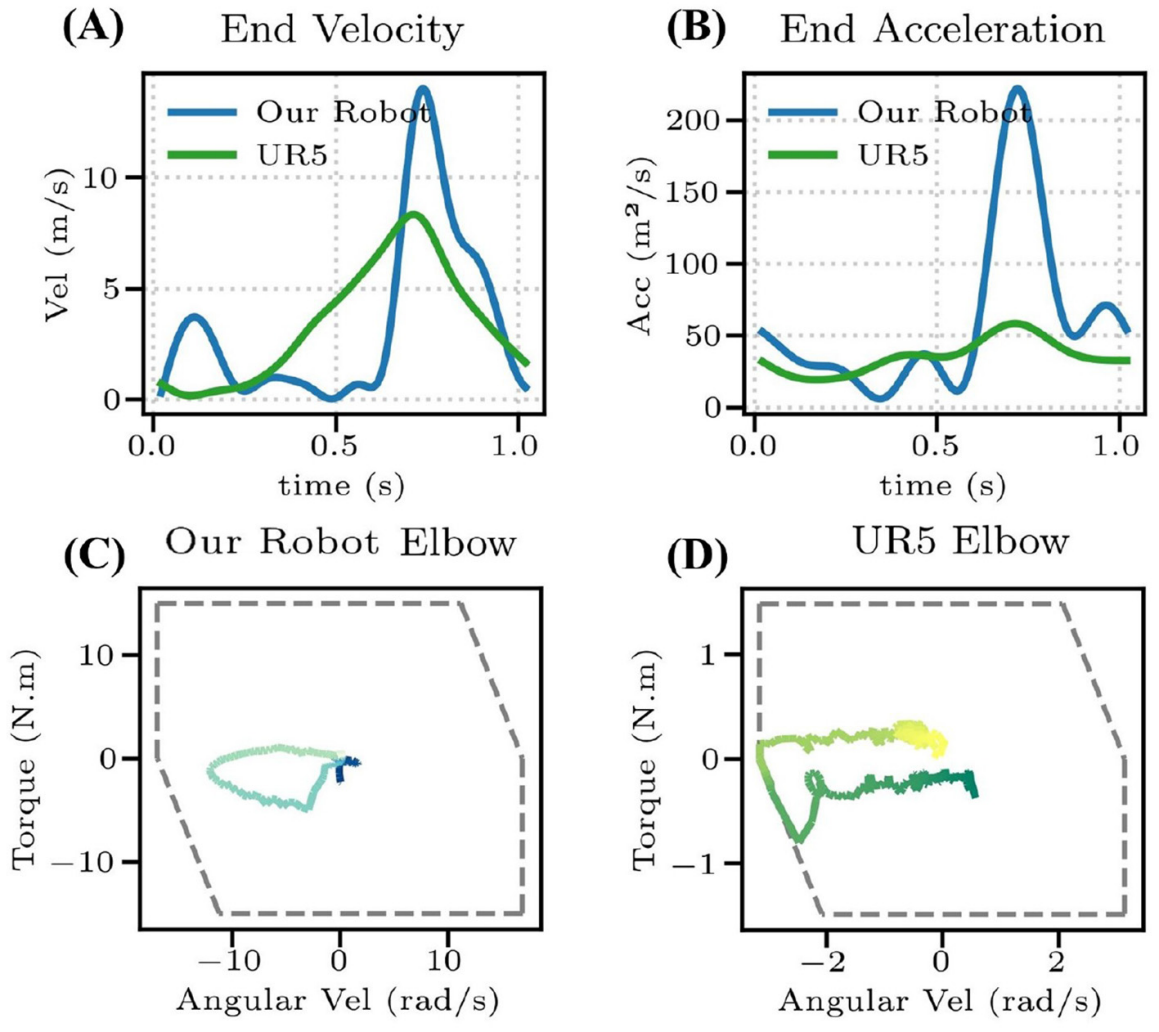
**(A)** The racket speed of KirinArm and UR5. **(B)** The racket acceleration of KirinArm and UR5. **(C, D)** Elbow torque-speed curve of the KirinArm and UR5, where the gray dashed line shows the motor constraint.

To investigate the reasons for the performance differences, we conducted a comparative analysis of both robotic arms' velocity and acceleration capabilities (Figure 11). KirinArm demonstrates superior terminal speed and acceleration, approximately twice and three times higher than those of UR5, respectively, explaining UR5's limitations in dynamic tasks. Torque-speed curves in Figures 11C, D reveal that UR5's elbow joint operates at its motor capacity limit (gray dashed lines are the motor constraints, which are modeled as a piecewise linear function to approximate the motors external characteristic curve Yuan et al., 2025), while KirinArm operates well within its limits. The limitation of UR5 is attributed to its higher mass, necessitating greater torque during rapid movements, and its high gear reduction ratio, limiting maximum joint speeds. The results demonstrate that dynamic capability is critical for high-speed badminton tasks.

### 3.7 Hardware experiment results

**Figure 12:**
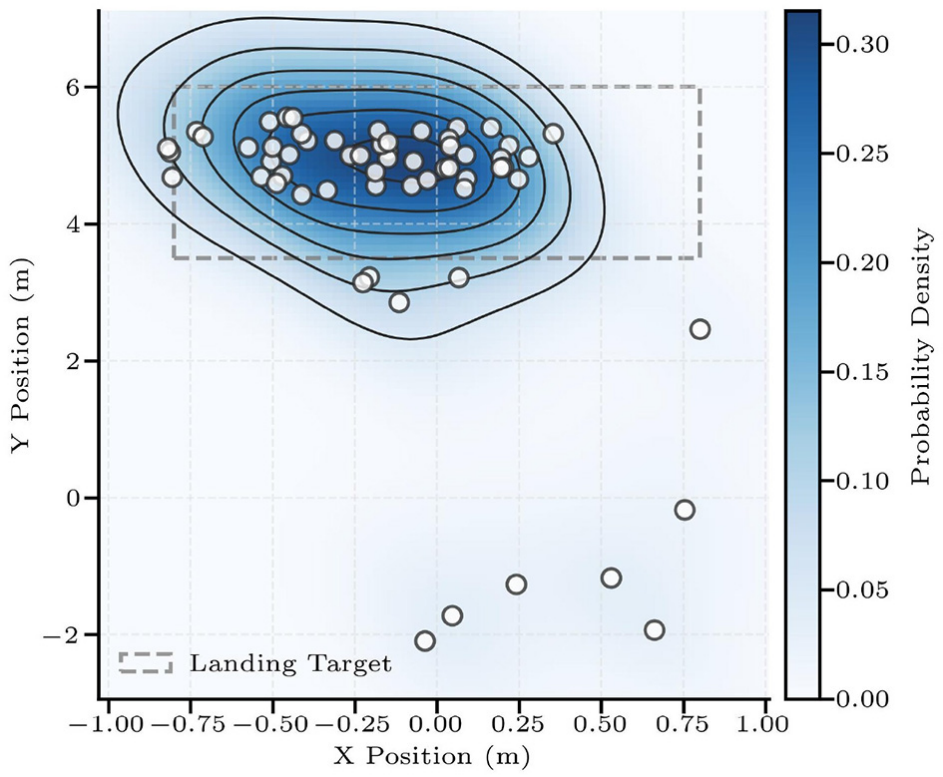
The shuttlecock landing points of 60 trials. The light gray area denotes the target area, while varying shades of blue represent the probability density of landings within that region.

Implemented on the hardware system, the framework achieves zero-shot transfer. Across 60 trials with randomized initial states of the shuttlecock (generated by a pan-tilt shuttlecock launcher), the DTG-IRRL controller achieved a 90% hitting rate (54/60) and a 70% landing accuracy (42/60). Missed strikes (10%) occurred only when the shuttlecocks altitude exceeded the arm's workspace. The landing positions showed a deviation of 0.2 m (Figure 12), confirming both the controller's spatial consistency and its practical applicability for badminton tasks.

**Figure 13:**
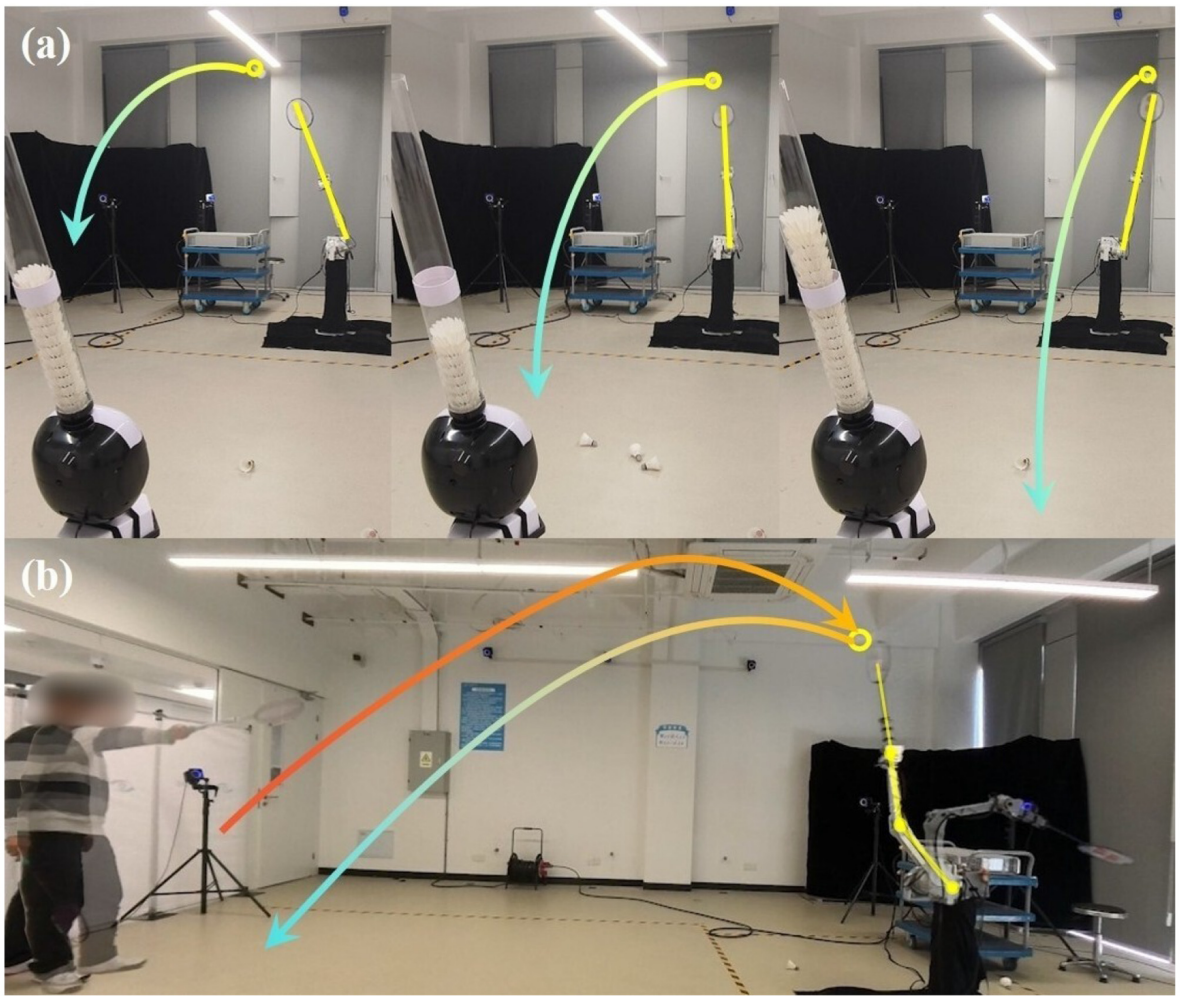
**(A)** Robotic arm successfully intercepting shuttlecocks from varied launch angles during robot-launcher interaction, and **(B)** multi-rally human-robot interaction trials. The gradient arrows depict the shuttlecock's trajectory, while the yellow segments represent the robotic arm's posture.

Human-robot interaction tests with three novice players (Figure 13) demonstrated sustained rally capability (an average of six consecutive hits), with physical implementation achieving simulation-equivalent performance while confirming real-world robustness. The experiment video is available in the Supplementary material and on the project website (https://stylite-y.github.io/DTG-IRRL-For-Badminton/).

## 4 Conclusion

In this study, we propose a learning framework (DTG-IRRL) for robotic badminton to address the convergence difficulties in RL due to sparse rewards and the trajectory prediction challenges posed by non-linear dynamics. The framework achieves zero-shot transfer on a robot system, demonstrating a 90% hitting rate, a 70% landing accuracy, and enabling sustained multi-round human-robot rallies. Further analysis of the reward function on a special hyperplane demonstrates that DTG-IRRL can effectively mitigate the challenges of local optima and slow convergence due to sparse rewards. Comparative experiments with UR5 confirm the framework's cross-platform generalization capability and highlight the importance of high dynamic performance for high-speed tasks. While the proposed framework demonstrates promising results, its performance is constrained by the absence of a mobile platform and a limited repertoire of badminton techniques. Future studies will integrate a mobile platform and expand the stroke techniques (such as smash, drop shot, and net shot) to achieve human-level badminton performance.

## Data Availability

The original contributions presented in the study are included in the article/Supplementary material, further inquiries can be directed to the corresponding authors.
